# Estimated Effectiveness of Influenza Vaccines in Preventing Secondary Infections in Households

**DOI:** 10.1001/jamanetworkopen.2024.46814

**Published:** 2024-11-21

**Authors:** Carlos G. Grijalva, Huong Q. Nguyen, Yuwei Zhu, Alexandra M. Mellis, Trey McGonigle, Jennifer K. Meece, Jessica E. Biddle, Natasha B. Halasa, Carrie Reed, Alicia M. Fry, Yang Yang, Edward A. Belongia, H. Keipp Talbot, Melissa A. Rolfes

**Affiliations:** 1 Vanderbilt University Medical Center, Nashville, Tennessee; 2 Marshfield Clinic Research Institute, Marshfield, Wisconsin; 3 CDC Influenza Division, Centers for Disease Control and Prevention, Atlanta, Georgia; 4 Fulton County Board of Health, Atlanta, Georgia; 5 University of Georgia, Atlanta

## Abstract

**Importance:**

Influenza vaccine effectiveness (VE) is commonly assessed against prevention of illness that requires medical attention. Few studies have evaluated VE against secondary influenza infections.

**Objective:**

To determine the estimated effectiveness of influenza vaccines in preventing secondary infections after influenza was introduced into households.

**Design, Settings, and Participants:**

During 3 consecutive influenza seasons (2017-2020), primary cases (the first household members with laboratory-confirmed influenza) and their household contacts in Tennessee and Wisconsin were enrolled into a prospective case-ascertained household transmission cohort study. Participants collected daily symptom diaries and nasal swabs for up to 7 days. Data were analyzed from September 2022 to February 2024.

**Exposures:**

Vaccination history, self-reported and verified through review of medical and registry records.

**Main Outcomes and Measures:**

Specimens were tested using reverse transcription-polymerase chain reaction to determine influenza infection. Longitudinal chain binomial models were used to estimate secondary infection risk and the effectiveness of influenza vaccines in preventing infection among household contacts overall and by virus type and subtype and/or lineage.

**Results:**

The analysis included 699 primary cases and 1581 household contacts. The median (IQR) age of the primary cases was 13 (7-38) years, 381 (54.5%) were female, 60 (8.6%) were Hispanic, 46 (6.6%) were non-Hispanic Black, 553 (79.1%) were Non-Hispanic White, and 343 (49.1%) were vaccinated. Among household contacts, the median age was 31 (10-41) years, 833 (52.7%) were female, 116 (7.3%) were Hispanic, 78 (4.9%) were non-Hispanic Black, 1283 (81.2%) were non-Hispanic White, 792 (50.1%) were vaccinated, and 356 (22.5%) had laboratory-confirmed influenza during follow-up. The overall secondary infection risk of influenza among unvaccinated household contacts was 18.8% (95% CI, 15.9% to 22.0%). The risk was highest among children and was 20.3% (95% CI, 16.4% to 24.9%) for influenza A and 15.9% (95% CI, 11.8% to 21.0%) for influenza B. The overall estimated VE for preventing secondary infections among household contacts was 21.0% (95% CI, 1.4% to 36.7%) and varied by type; estimated VE against influenza A was 5.0% (95% CI, −22.3% to 26.3%) and 56.4% (95% CI, 30.1% to 72.8%) against influenza B.

**Conclusions and Relevance:**

After influenza was introduced into households, the risk of secondary influenza among unvaccinated household contacts was approximately 15% to 20%, and highest among children. Estimated VE varied by influenza type, with demonstrated protection against influenza B virus infection.

## Key Points

**Question:**

What is the estimated effectiveness of influenza vaccines in preventing secondary infections after influenza is introduced into households?

**Findings:**

In this case-ascertained cohort study of 699 primary cases and 1581 household contacts, the secondary infection risk of influenza infection among unvaccinated household contacts was 18.8% (95% CI, 15.9% to 22.0%). The estimated effectiveness of influenza vaccines for preventing secondary infections among household contacts was 21.0% (95% CI, 1.4% to 36.7%).

**Meaning:**

Influenza vaccination was associated with a reduced risk of laboratory-confirmed influenza virus infection among household contacts.

## Teaser

This cohort study evaluates the estimated effectiveness of influenza vaccines in preventing secondary infections after influenza is introduced into households.

## Introduction

Households, where individuals often interact in close contact, provide a favorable environment for transmission of respiratory viruses. Previous household studies indicated that 3% to 38% of household members would be infected after a first member was infected with influenza and identified school-aged children as important contributors to influenza transmission in households.^1,2,3,4,5,6,7^ Yet, most of those seminal studies were conducted several years ago or during the 2009 pandemic, and more recent prospective assessments of transmission of influenza in US households are scarce.

Influenza vaccines are recommended in the US for individuals aged 6 months and older for the prevention of influenza and related complications. While the effectiveness of influenza vaccines in preventing medically attended illness and serious disease leading to hospitalization has been demonstrated and is monitored closely,^8,9,10,11,12^ the effectiveness of influenza vaccines in preventing infections regardless of symptoms remains unclear and is uncommonly studied.^13^ The degree to which vaccines reduce secondary infections after exposure to influenza, especially in high transmission settings, like households, may be especially informative to individuals at increased risk for severe influenza complications.^14^

In collaboration with the US Centers for Disease Control and Prevention (CDC), we conducted the Influenza Transmission Evaluation Study (FLUTES), a case-ascertained household transmission study at 2 research sites during 3 consecutive influenza seasons in the US. We assessed the incidence of laboratory-confirmed influenza virus infection in household members with the specific objective of estimating secondary infection risks and the effectiveness of influenza vaccines in preventing secondary infections in household contacts.

## Methods

### Study Design

Over 3 consecutive influenza seasons (2017-2020), we identified and enrolled individuals with laboratory-confirmed influenza virus infection who presented for ambulatory care at walk-in-clinics in middle Tennessee and outpatient clinics in central Wisconsin. Individuals were considered primary cases if they were the first person in the household to become ill. Primary cases were eligible for this analysis if their clinical disease started within the previous 5 days of clinical testing, and if they lived with at least 1 other household member who had no symptoms compatible with influenza on the primary case’s disease onset date. The primary case and their household contacts provided written consent and enrolled within 7 days of the primary case’s disease onset and followed up daily for up to 7 days. We enrolled new study households during each influenza season at each site, which was defined by local influenza surveillance.^15^ Study protocols and procedures were reviewed by the institutional review boards at the Marshfield Clinic Research Institute and Vanderbilt University Medical Center. The CDC determined these activities were conducted consistent with applicable federal law and CDC policy (see 45 CFR part 46; 21 CFR part 56). This study followed the Strengthening the Reporting of Observational Studies in Epidemiology (STROBE) reporting guideline.

### Data Collection

At enrollment, we collected sociodemographic information and data on household characteristics (including self-reported race and ethnicity [Hispanic, non-Hispanic Black, non-Hispanic White, and Other/refused]; assessed because it has been previously associated with risk of infection), medical history, and influenza vaccination history using electronic or paper-based surveys customized according to participants’ preference. During each day of follow-up, participants reported symptoms and use of medications commonly used for infection symptoms (eg, antipyretics). Study forms were available in English and Spanish. Study data were collected and securely stored in dedicated Research Electronic Data Capture (REDCap) databases.^16^

### Specimen Collection

For collection of specimens, we conducted an initial enrollment household visit, with nasal swab specimens collected from index cases and household contacts by trained research staff while demonstrating self- and/or parent collection of nasal swab specimens and storage procedures to household members. After the enrollment visit, participants and/or parents collected nasal swab specimens daily, regardless of the presence of symptoms, throughout follow-up. Nasal swabs were collected in viral transport media and refrigerated in closed containers at participants’ homes. Collected specimens were retrieved by the study team during follow-up visits every 3 to 5 days and transported to the study research laboratories for testing. For participants who preferred to meet with research staff outside their households, meetings at clinics or other convenient locations were also conducted.

### Identification of Influenza Virus Infections

Collected specimens were tested for influenza using real-time reverse transcription polymerase chain reaction (RT-PCR) using CDC-approved protocols and primers^17^ at research laboratories at Vanderbilt University Medical Center and Marshfield Clinic Research Institute. To ensure integrity of the specimens, the presence of human RNaseP, a housekeeping gene, was verified. Detected influenza viruses had type and subtype and/or lineage determined from study specimens using RT-PCR or available clinical records, as previously reported.^12,18^ Test results with a cycle threshold of 40 or less were considered positive for the specific reaction target, and results with a cycle threshold greater than 40 were considered negative. Laboratory personnel conducting the RT-PCR testing were blinded to the participants’ vaccination status. Individuals were considered to be infected with influenza virus if they had at least 1 specimen with a positive test result.

### Vaccination History and Verification

Self-reported influenza vaccination history was collected, reviewed, verified, and categorized using available medical records, state vaccination registries, or other sources of vaccination data, using methods previously described.^8,9,10,12^ Participants were considered vaccinated for the current season if they had received an influenza vaccination that was verified after record review, or a vaccination where a valid date and location of vaccination were reported, at least 14 days before the primary case’s disease onset date.

### Statistical Analysis

The analytic population included participants with complete vaccination information, at least 3 collected specimens with laboratory results available, and (if symptomatic) nonmissing dates of symptom onset. In addition, we excluded households where we could not identify a primary case, those households where no influenza virus infection was demonstrated in a primary case by molecular research testing, and households that had more than 1 influenza type, subtype, or lineage detected.

We first characterized the primary cases and their household contacts using descriptive statistics. Serial intervals were estimated by subtracting the primary case’s disease onset date from the disease onset date of household contacts with influenza. To estimate the secondary infection risk among household contacts after influenza virus infection was introduced in their household, we applied a longitudinal chain binomial model,^19,20,21^ which was designed for the assessment of transmission dynamics of infectious diseases within close contact groups such as households and takes into account the probability of transmission from multiple primary cases; probability of tertiary, or subsequent transmissions within the household; and the probability of infection by nonspecific sources outside the household (see eMethods, eTable 1, and eFigure in Supplement 1). Briefly, for each day of follow-up, we estimated transmission probabilities between each possible infector and susceptible, uninfected household member, informed by assumptions about the incubation period (peaking at 2 days postinfection)^22^ and duration of shedding (peaking at disease onset and declining until day 7 postonset)^23^ of influenza virus. These models estimated the probabilities of acquiring infection accounting for the age of the contact, vaccination status of the contact, season, and household size. We also compared the odds of laboratory-confirmed influenza virus infection between vaccinated and unvaccinated household contacts adjusted for potential confounders including age of contact (<5, 5-17, 18-49, or ≥50 years), household size (number of household members), and season (2017-2018, 2018-2019, or 2019-2020), computing adjusted odds ratios (aOR). Estimated vaccine effectiveness (VE) against infection was calculated as 1 – aOR, and the overall VE for influenza was calculated from a model without distinguishing influenza types. The overall VEs for influenza A and influenza B, respectively, were estimated using a joint model for the 2 types. We then applied models to each influenza type, where VE was further stratified by subgroups including subtype and/or lineage, site, season, and age group. The type-specific overall VE and subgroup-specific VE estimates were derived from appropriate interaction term(s) between vaccination status and the corresponding type or subgroup indicator. All tests were 2-sided and a *P *value less than .05 was considered significant. Statistical analyses were conducted in R version 4.4.0 (R Project for Statistical Computing) and TranStat (TranStat).^24^ Data were analyzed from September 2022 to February 2024.

## Results

### Study Population

We prospectively enrolled 708 households encompassing 2382 participants from 2017 to 2020. After application of selection criteria, the analysis included 677 households (96%) and 2280 participants (96%), encompassing 699 primary or coprimary cases and 1581 household contacts (Figure). The median (IQR) age of the primary cases was 13 (7-38) years, 381 (54.5%) were female, 60 (8.6%) were Hispanic, 46 (6.6%) were non-Hispanic Black, and 553 (79.1%) were non-Hispanic White. The median (IQR) age of the household contacts was 31 (10-41) years, 833 (52.7%) were female, 116 (7.3%) were Hispanic, 78 (4.9%) were non-Hispanic Black, 1283 (81.2%) were non-Hispanic White (Table 1).

**Figure.**
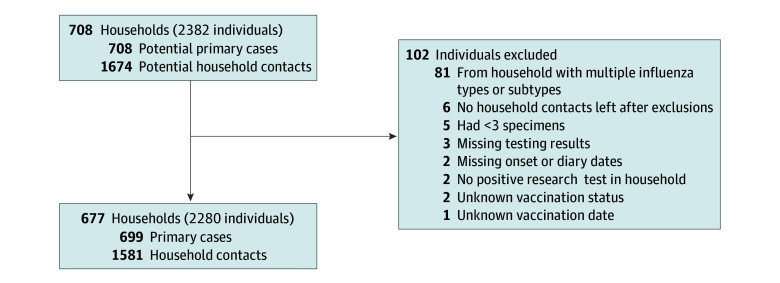
Flowchart of Enrollment, Influenza Transmission Evaluation Study, Middle Tennessee and Central Wisconsin, 2017 to 2020

**Table 1.** Characteristics of Primary Cases and Household Contacts, Influenza Transmission Evaluation Study, Middle Tennessee and Central Wisconsin, 2017 to 2020

Characteristic	Participants, No. (%)		
	Primary cases (n = 699)	Household contacts (n = 1581)	All participants (N = 2280)
Age, median (IQR), y	13 (7-38)	31 (10-41)	23 (9-41)
Age group, y			
<5	73 (10.4)	133 (8.4)	206 (9.1)
5-17	337 (48.2)	515 (32.6)	852 (37.4)
18-49	180 (25.8)	740 (46.8)	920 (40.4)
≥50	109 (15.6)	193 (12.2)	302 (13.2)
Gender^a^			
Women	381 (54.5)	833 (52.7)	1214 (53.2)
Men	318 (45.5)	744 (47.1)	1062 (46.6)
Race and ethnicity			
Hispanic	60 (8.6)	116 (7.3)	176 (7.7)
Non-Hispanic Black	46 (6.6)	78 (4.9)	124 (5.4)
Non-Hispanic White	553 (79.1)	1283 (81.2)	1836 (80.5)
Other/refused^b^	40 (5.7)	104 (6.6)	144 (6.3)
Vaccinated	343 (49.1)	792 (50.1)	1135 (49.8)
Enrolling site			
Central Wisconsin	331 (47.4)	870 (55.0)	1201 (52.7)
Middle Tennessee	368 (52.6)	711 (45.0)	1079 (47.3)
Enrolling season			
2017-2018	162 (23.2)	318 (20.1)	480 (21.0)
2018-2019	236 (33.8)	568 (35.9)	804 (35.3)
2018-2019	301 (43.1)	695 (44.0)	996 (43.7)

^a^ Four participants refused to report gender.

^b^ Indicates participants who chose other race or ethnicity.

### Secondary Infection Risk Among Household Contacts

The dominant influenza subtypes and/or lineages varied by season. During the 2017 to 2018 season, influenza A(H3N2) and B/Yamagata were the most common viruses detected; during the 2018 to 2019 season, most detections were influenza A(H3N2) virus; and during the 2019 to 2020 season, most detections were influenza A(H1N1)pdm09 and influenza B/Victoria viruses.

A total of 356 household contacts (22%) had a laboratory-confirmed influenza virus infection during follow-up, and 24 infected contacts (7%) were asymptomatic. The median (IQR) serial interval was 3 (2-4) days. Among influenza detections in household contacts, 267 (75%) were influenza A and 89 (25%) were influenza B virus. Overall, the chain binomial model-based secondary infection risk of laboratory-confirmed influenza virus infection among household contacts in the absence of vaccination was 18.8% (95% CI, 15.9 to 22.0%) during the study period. The secondary infection risk varied with age and was highest among children aged less than 5 years (20.3%; 95% CI, 16.4% to 24.9% for influenza A and 15.9%; 95% CI, 11.8% to 21.0% for influenza B) (Table 2). The risk of secondary infection also varied by the virus type and subtype and/or lineage the primary case was infected with, as well as across seasons, but was similar between study sites and according to household size (Table 2).

**Table 2.** Number of Infections Among Household Contacts and Secondary Infection Risk in the Absence of Vaccination, Influenza Transmission Evaluation Study, Middle Tennessee and Central Wisconsin, 2017 to 2020^a^

Infection or Characteristic	No. of laboratory-confirmed infections/household contacts	Secondary infection risk (95% CI)
Overall	356/1581	18.8 (15.9-22.0)
Influenza type		
Influenza A	267/1020	20.3 (16.4-24.9)
A(H1N1)pdm09	160/506	21.6 (15.9-28.5)
A(H3N2)	106/423	18.1 (13.0-24.7)
Influenza B	89/561	15.9 (11.8-21.0)
B (Victoria)	78/340	17.6 (11.5-26.0)
B (Yamagata)	11/95	11.3 (5.5-21.8)
Age group, y		
<5	48/133	29.9 (23.0-37.8)
5-17	139/515	23.3 (19.0-28.1)
18-49	128/740	14.0 (11.4-17.2)
≥50	41/193	20.4 (14.7-27.4)
Household size		
2	45/219	21.6 (15.8-28.7)
3	71/329	20.3 (15.6-25.9)
≥4	240/1033	18.3 (15.4-21.5)
Site		
Central Wisconsin	216/870	19.6 (16.4-23.2)
Middle Tennessee	140/711	17.1 (13.6-21.1)
Season		
2017-2018	44/318	12.4 (8.9-16.9)
2018-2019	130/568	18.4 (14.8-22.5)
2019-2020	182/695	20.9 (17.3-25.1)

^a^ Secondary infection risks by influenza type and/or subtype, age group, household size, site, and season were estimated using chain binomial models accounting for vaccination. All estimates for secondary infection risks derived for unvaccinated contacts. Household contacts with detected influenza infection but without subtype and/or lineage information were not included in corresponding subtype and/or lineage estimates.

### VE Against Infection Among Household Contacts

The overall effectiveness of influenza vaccines in preventing laboratory-confirmed infections, across all influenza types, among household contacts during the 3 seasons was estimated to be 21.0% (95% CI, 1.4% to 36.7%); however, VE estimates varied substantially by influenza type and season (Table 3). Estimated VE for preventing influenza B among household contacts was demonstrated (56.4%; 95% CI, 30.1% to 72.8%), especially among those aged 5 to 17 years (88.4%; 95% CI, 75.1% to 94.6%) and those aged 18 to 49 years (70.8%; 95% CI, 28.5% to 88.1%). The VE against influenza B (Victoria) and B (Yamagata) virus infections was estimated as 49.5% (95% CI, 14.1% to 70.3%) and 25.5% (95% CI, −162.1% to 78.8%), respectively (eTable 3 in Supplement 1).

**Table 3.** Vaccine Effectiveness Against Influenza Infection Among Household Contacts, Influenza Transmission Evaluation Study, Middle Tennessee and Central Wisconsin, 2017 to 2020^a^

Characteristic	Influenza A			Influenza B		
	Proportion of contacts infected, No./total No. (%)		Adjusted vaccine effectiveness (95% CI)	Proportion of contacts infected, No./total No. (%)		Adjusted vaccine effectiveness (95% CI)
	Vaccinated	Unvaccinated		Vaccinated	Unvaccinated	
Overall	134/534 (25.1)	133/486 (27.4)	5.0 (−22.3 to 26.3)	25/258 (9.7)	64/303 (21.1)	56.4 (30.1 to 72.8)
Age group						
<5 y	20/49 (40.8)	18/36 (50.0)	34.6 (−18.5 to 63.9)	6/28 (21.4)	4/20 (20.0)	−10.7 (−306.7 to 69.9)
5-17 y	39/165 (23.6)	59/183 (32.2)	23.8 (−18.7 to 51.1)	9/66 (13.6)	32/101 (31.7)	88.4 (75.1 to 94.6)
18-49 y	50/234 (21.4)	46/222 (20.7)	−13.1 (−71.6 to 25.5)	6/124 (4.8)	26/160 (16.3)	70.8 (28.5 to 88.1)
≥50 y	25/86 (29.1)	10/45 (22.2)	−44.9 (−210 to 32.3)	4/40 (10.0)	2/22 (9.1)	−58.7 (−798 to 71.9)
Site						
Central Wisconsin	73/270 (27.0)	85/304 (28.0)	0 (−39.1 to 28.2)	13/115 (11.3)	45/181 (24.9)	59.7 (24.4 to 78.5)
Middle Tennessee	61/264 (23.1)	48/182 (26.4)	16.5 (−23.7 to 43.7)	12/143 (8.4)	19/122 (15.6)	44.0 (−18.8 to 73.6)
Season						
2017-2018	16/96 (16.7)	16/77 (20.8)	21.6 (−61.4 to 62)	5/69 (7.2)	7/76 (9.2)	14.7 (−175.9 to 73.6)
2018-2019	71/258 (27.5)	58/243 (23.9)	−14.0 (−63.6 to 20.6)	0/41	1/26 (3.8)	NA^b^
2019-2020	47/180 (26.1)	59/166 (35.5)	24.4 (−13 to 49.5)	20/148 (13.5)	56/201 (27.9)	57.8 (28.2 to 75.1)

Abbreviation: NA, not applicable.

^a^ Overall influenza type and/or subtype-specific vaccine effectiveness estimates were estimated using longitudinal chain binomial models and accounted for age group, site, household size, and season as appropriate. Other vaccine effectiveness estimates accounted for the other variables included in the table, as appropriate.

^b^ Indicates estimates cannot be obtained due to 0 events.

No detectable VE was found for preventing influenza A virus infections during the study seasons (5.0%; 95% CI, −22.3% to 26.3%). The VE against influenza A(H1N1)pdm09 and A(H3N2) was estimated as 21.4% (95% CI, −9.2% to 43.5%) and −26.9% (95% CI, −91.0% to 15.6%), respectively (eTable 2 in Supplement 1).

## Discussion

In this prospective case-ascertained household transmission study conducted at 2 different US sites during 3 consecutive influenza seasons with daily collection of data and respiratory specimens following introduction of infections in the households, we observed a substantial risk of subsequent influenza virus infection among household contacts. Approximately 1 in 5 household contacts became infected during follow-up, with the highest risk of infection observed among children. The risk of secondary infection varied across seasons. The prospective case-ascertained household transmission study design we used, with systematic daily testing of household contacts regardless of symptoms, allowed for estimation of VE against PCR-confirmed influenza virus infection. We observed a reduction in infection risk among vaccinated contacts. Influenza type-specific protection was demonstrated against influenza B infections, especially among children aged older than 5 years and younger than 50 years. No protection was demonstrated against infection with influenza A viruses.

The estimates of VE against any influenza virus infection in the present study were lower than those generated from surveillance of medically attended illness. The CDC influenza VE network estimated that the overall effectiveness of vaccines in preventing medically attended influenza was 38%, 29%, and 39% in the 2017 to 2018, 2018 to 2019, and 2019 to 2020 seasons, respectively. Our observations of relatively modest VE against infection could suggest that vaccines confer different degrees of protection against infection than more severe outcomes, or that VE is lower in close contact environments like households.^3^ Vaccines can confer higher protection against severe outcomes like hospitalization than against outpatient visits^25^ and may confer better protection against severe outcomes during years of close antigenic match between the circulating virus and the vaccine strain.^26^ While case-ascertained studies are capable of efficiently^4,7,27,28^ estimating VE against any infection,^13^ influenza exposure is more intense in household than community settings. Previous cohorts have demonstrated VE against symptomatic laboratory-confirmed influenza acquired from community but not household settings.^3^ These observations are consistent with a leaky vaccine framework that partially reduces probability of infection on each exposure.^29^ If VE is lower in the household setting than in community settings, these estimates of VE against infection may approach a lower bound of VE against any infection in the general population.

Influenza vaccination is the primary strategy to prevent influenza and its related complications.^30^ Estimating the effect of vaccination on the risk of subsequent influenza infections among household contacts informs our understanding of how infections transmit. Lower VE in household settings suggests that additional measures could be considered to prevent the spread of influenza once it is introduced into the household if the goal is to prevent secondary infections. These considerations may be especially important for household members at increased risk for influenza complications, including young children, pregnant people, adults aged 65 years or older, and people with certain chronic medical conditions. Complementary preventive measures could include isolation of ill household members, improved ventilation, hand hygiene, disinfection of surfaces, use of masks and covering coughs and sneezes, and antiviral prophylaxis.^31^

We observed varied VE by influenza type, subtype, and season. This is consistent with prior findings that VE point estimates for prevention of influenza A(H3N2) have been lower than those against influenza A(H1N1)pdm09, or influenza B.^32,33,34^ Circulation of drifted influenza A(H3N2) and A(H1N1)pdm09 viruses not antigenically matched to seasonal vaccines and related lower VE were documented during the study seasons,^35^ and the overall VE estimated in this study may not be applicable to other seasons. Specifically, VE against influenza A may be higher in seasons of closer antigenic match than those seasons included in this study. The variation we observed by season underscores broader seasonal variability in the effectiveness of influenza vaccines, which have their composition revised almost every season in an attempt to match the future circulating virus strains as they continue to evolve.

In addition to estimating VE, this study provides estimates of the household secondary infection risks of influenza, which are uncommonly estimated in the US. During the 2009 pandemic, estimates that relied on self-reported symptoms without laboratory confirmation estimated that about 13% and 10% of household contacts experienced acute respiratory and influenza-like illness, respectively.^36^ In contrast, this study with systematic daily testing of household contacts, regardless of symptoms, demonstrated higher secondary infection risks during influenza seasons. Thus, this study design provides a more comprehensive identification of secondary infections^4^ than, for example, weekly assessments or symptom-triggered specimen collection.^3,7,27,37,38^ In this study, about 7% of detected secondary infections among household contacts were asymptomatic, consistent with recent reports that highlight this important occurrence.^39,40^

### Strengths and Limitations

This study has several strengths that mitigate common limitations to the estimation of VE against infection,^13^ including rapid enrollment of households,^6,35^ systematic daily collection of data and specimens, extensive efforts to verify vaccination information, molecular determination of viral infections using highly sensitive molecular techniques robust to differences in specimen collection between study staff and participants,^41^ and application of advanced analytics to enable the identification and quantification of transmission events.^42^ However, our findings must be interpreted considering several limitations. First, recruitment of index cases from clinical testing pools may limit the generalizability of these secondary infection risks to households where the primary case had a more mild or asymptomatic infection.^39,40,43^ Second, we did not collect acute and convalescent serum specimens to supplement detection of infections of systematically collected respiratory specimens or to assess the role of baseline immunological status on the susceptibility to infection. Third, we were not able to assess the effectiveness of specific vaccine formulations (eg, high-dose vaccines). The impact of this limitation, however, is mitigated by the small number of older adults eligible to receive high-dose or adjuvanted vaccines in this study. Fourth, although the total number of recruited households was large, stratification of estimates by influenza subtypes and lineages was challenging due to small cell sizes, which limited the precision of some estimates. In addition, while we observed similar patterns of transmission in our different study locations, our observations may not be directly generalizable to other geographic settings. Moreover, our results are based on an observational study, and although our analyses accounted for relevant measured confounders, we cannot rule out residual confounding. This report did not examine the impact of vaccination of possible infectors to reduce onwards transmission, which would require a larger study. The extent to which the observed protection is mediated by specific immune responses to vaccination (for example, mucosal vs cellular or humoral immunity, overall age, and age-specific immune histories) requires further study.

## Conclusion

Our study showed that following introduction of influenza virus infections in households, there is a high risk of transmission to household members. Children were commonly identified as primary cases, and contact household children experienced the highest risk of secondary infection. During the study period, influenza vaccination was associated with a reduced risk of laboratory-confirmed influenza virus infection, especially influenza B virus. Although complementary preventive strategies to prevent influenza in household settings may be considered, seasonal influenza vaccination is the primary strategy recommended for prevention of influenza illness and its complications.

## Notes

Supplement 1.
eMethods.
**eFigure. **Assumed Incubation Period and Infectivity Profiles **eTable 1. **Estimates of Baseline b and p for Various Vaccine Effectiveness Models **eTable 2. **Vaccine Effectiveness Against Influenza A Infection Among Household Contacts, FluTES, Middle Tennessee and Central Wisconsin (2017-2020) **eTable 3.** Vaccine Effectiveness Against Influenza B Infection Among Household Contacts, FluTES, Middle Tennessee and Central Wisconsin (2017-2020)

Supplement 2.
Data Sharing Statement
