# Errors in Abstract Results

**DOI:** 10.1001/jamanetworkopen.2024.59710

**Published:** 2025-01-15

In the Original Investigation titled “Estimated Effectiveness of Influenza Vaccines in Preventing Secondary Infections in Households,”^1^ published November 21, 2024, there were errors in the Abstract Results. The Abstract Results stated that the overall secondary infection risk of influenza among household contacts was 18.8%, but it should have stated that the overall secondary infection risk of influenza among unvaccinated household contacts was 18.8%. In addition, the Abstract Results stated the overall estimated VE for preventing secondary infections among unvaccinated household contacts was 21.0%, but it should have stated that the overall estimated VE for preventing secondary infections among household contacts was 21.0%. This article has been corrected.^1^
