# Supplementary material for: Estimated Effectiveness of Influenza Vaccines in Preventing Secondary Infections in Households
Source: JAMA Netw Open. 2024 Nov 21;7(11):e2446814. doi: 10.1001/jamanetworkopen.2024.46814 (PMC11582933; doi:10.1001/jamanetworkopen.2024.46814)
Supplement: Supplement 1. — eMethods. eFigure. Assumed Incubation Period and Infectivity Profiles eTable 1. Estimates of Baseline b and p for Various Vaccine Effectiveness Models eTable 2. Vaccine Effectiveness Against Influenza A Infection Among Household Contacts, FluTES, Middle Tennessee and Central Wisconsin (2017-2020) eTable 3. Vaccine Effectiveness Against Influenza B Infection Among Household Contacts, FluTES, Middle Tennessee and Central Wisconsin (2017-2020) [file jamanetwopen-e2446814-s001.pdf]

Supplemental Online Content

Grijalva CG, Nguyen HQ, Zhu Y, et al. Estimated effectiveness of influenza vaccines in preventing secondary infections in households. *JAMA Netw Open*. 2024;7(11):e2446814. doi:10.1001/jamanetworkopen.2024.46814

eMethods.

eFigure. Assumed Incubation Period and Infectivity Profiles

eTable 1. Estimates of Baseline b and p for Various Vaccine Effectiveness Models

eTable 2. Vaccine Effectiveness Against Influenza A Infection Among Household Contacts, FluTES, Middle Tennessee and Central Wisconsin (2017-2020)

eTable 3. Vaccine Effectiveness Against Influenza B Infection Among Household Contacts, FluTES, Middle Tennessee and Central Wisconsin (2017-2020)

This supplemental material has been provided by the authors to give readers additional information about their work.

eMethods

Chain binomial model

A longitudinal chain binomial model implemented in discrete time was used to estimate transmission probabilities, which has been developed and described elsewhere. On each day t, there is a probability of transmission both within household groups, between infectious and susceptible household members, and a probability of transmission from general contact with other community members and each susceptible member of the household. Let each household h be represented as a set of household members Λ_h. The baseline daily probability of transmission is given as p, such that at time t the effective transmission probability transmission from an infectious person i to a susceptible person j in their household is:

$$p_{ij}(t) = \mathrm{logit}(p) + \beta' x_{ij}(t)$$

Where β is the coefficient vector corresponding to covariates $x_{ij}(t)$. At this same time t, the effective probability of infection due to contact with the general public is:

$$b_j(t) = \mathrm{logit}(b) + a' x_j(t)$$

where b is the baseline probability of infection from external contact, and α is the coefficient vector corresponding to covariates $x_j(t)$. As only covariates of susceptible contacts were represented in both $x_{ij}(t)$ and $x_j(t)$, we can assume the regression coefficients a and β are the same and use β to denote both. This assumption implies that, for example, vaccination has the same impact on risk of acquiring an infection inside of the home as it does on acquiring an infection from the unobserved community members. Let $\tilde{t}_i$ be the date of symptom onset if person i has a symptomatic infection, and we assume symptom onset also marks infectiousness onset, i.e., the incubation period and latent period coincide with each other. For asymptomatic infections, we use $\tilde{t}_i$ to indicate the day of infectiousness onset. For uninfected contacts, $\tilde{t}_i$ is undefined or we can let $\tilde{t}_i = \infty$. We assume the latent (incubation) period, $\tilde{t}_i - t_i$, where t_i is the infection day, follows a known discrete distribution, $\eta(\tilde{t}_i - t_i)$, regardless of whether i is symptomatic or asymptomatic. While the baseline transmission probability p is unknown, we assume the relative infectivity of each infected individual i over a fixed maximum duration of infectious period is known and denote it by $\phi(t - \tilde{t}_i)$, i.e., how much more (or less) infectious person i is on day t compared to the symptom (or infectiousness) onset day $\tilde{t}_i$. For influenza, $\phi(t - \tilde{t}_i)$ usually takes values between 0 and 1, and we set $\phi(0) = 1$, which means symptom onset also marks the peak infectivity. The exact choice of $\phi(t - \tilde{t}_i)$ is discussed later.

For a susceptible individual j in household h, the probability of escaping infection on day t from both the community source of infection and all infectious individuals in the same household is given by:

© 2024 Grijalva CG et al. *JAMA Network Open*.

$$e_j(t) = \left(1 - b_j(t)\right) \sum_{i \in \Lambda_h} \left[1 - \phi(t - \tilde{t}_i)\theta^{1-s_i} p_{ij}(t)\right],$$

where Λ_h is the collection of all household members. s_i indicates the symptom status of person i (1=symptomatic, 0=asymptomatic), and θ measures the relative infectivity of an asymptomatic infection compared to a symptomatic case. For simplicity, we assume $\theta = 1$, i.e., asymptomatic infections and symptomatic cases are equally infectious. The probability of an individual j escaping all infections from all possible sources up to day t is:

$$Q_j(t) = \prod_{\tau=1}^{t} e_j(\tau)$$

Let d_{min} and d_{max} are the minimum and maximum durations of the incubation (latent) period. Let $\tilde{t}_h = \{\tilde{t}_j : j \in \Lambda_h\}$ be the collection of all symptom/infectiousness onset days in household h, which represent the exposure history of all household members. The likelihood contributed by person i is then given by

$$L_{(i)}(b, p, \beta | \tilde{t}_h) = \begin{cases} Q_i(T_h), & \text{if uninfected,} \\ \sum_{t=\tilde{t}_i - d_{max}}^{\tilde{t}_i - d_{min}} \{\eta(t - \tilde{t}_i - d_{max})[1 - e_i(t)]Q_i(t-1)\}, & \text{if infected,} \end{cases} \quad (1)$$

where T_h is the last observation day of household h. As all household clusters must have at least one primary case, primary case infections do not contribute to the likelihood calculations, although exposure histories of other household members during a primary case's infectious period do.

Asymptomatic infections

Unlike symptomatic cases, we do not observe $\tilde{t}_i$ (infectiousness onset day) for asymptomatic infections, and such uncertainty need to be accounted for in the statistical inference. The exact approach used by our chain-binomial model to account for uncertainty associated with $\tilde{t}_i$ for asymptomatic cases has been described in detail elsewhere[20]. Briefly, a Monte Carlo Expectation-Maximization (MCEM) algorithm was used to sample $\tilde{t}_h$ over its all possible values, which is essentially determined by combinations of possible infectiousness onset days of asymptomatic infection in the household. The search space for possible days of infectiousness onset for a lab-confirmed asymptomatic individual was bounded by several assumptions. The earliest possible day was assumed to be the date of symptom onset of the primary case, to account for the possibility that asymptomatic individuals could have been co-primary cases. The latest possible day was assumed to be the collection day of the first influenza-positive specimen from that person.

Assumptions about the incubation period and relative infectivity profiles

Both $\eta(\tilde{t}_i - t)$, the distribution of the incubation period, and $\phi(t - \tilde{t}_i)$, the relative infectivity profile over the infectious period, were assumed to be discrete. $\eta(\tilde{t}_i - t)$

© 2024 Grijalva CG et al. *JAMA Network Open*.

corresponds to a discrete probability mass function ranging between 1 and 3 days, with mean of 2 days (Lessler et al., 2009). As Infectivity was assumed to peak at the date of symptom onset (or infectiousness onset for an asymptomatic infection), $\phi(t - \tilde{t}_i)$ takes value 1 when $t = \tilde{t}_i$ and gradually declines towards 0 until 7 days post-onset, in accordance with historical viral load data (Lau et al., 2010). See eFigure 1 for $\eta(\tilde{t}_i - t)$ and $\phi(t - \tilde{t}_i)$ used in the analysis.

© 2024 Grijalva CG et al. *JAMA Network Open*.

eFigure. Assumed Incubation Period and Infectivity Profiles

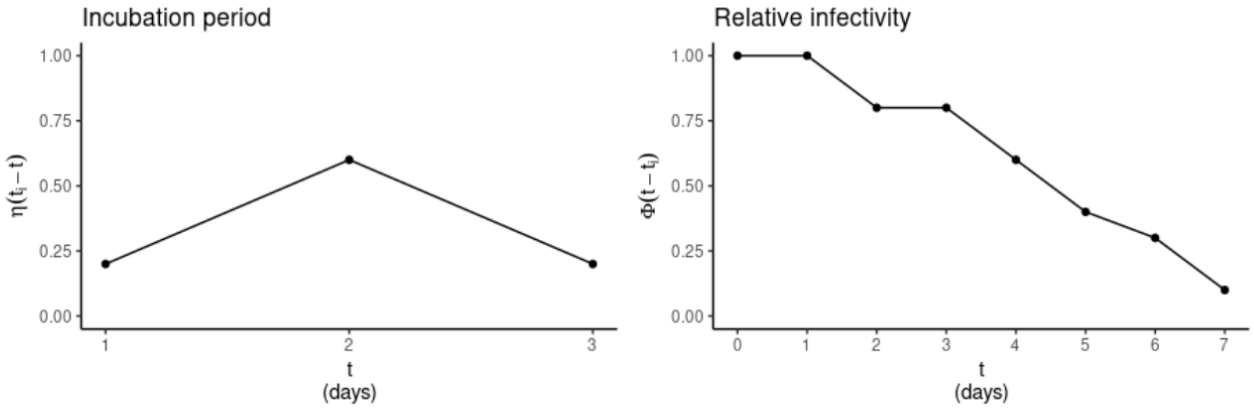

Footnote: The incubation period of influenza represents the time from infection to symptom onset (or peak infectivity, for asymptomatic cases); the relative infectivity represents the infectivity remaining for each day since peak infectivity. For symptomatic cases, peak infectivity was assumed to be the day of symptom onset.

© 2024 Grijalva CG et al. *JAMA Network Open*.

eTable 1. Estimates of Baseline b and p for Various Vaccine Effectiveness Models

**Model**	**b**	**p**
Overall, VE	0.0017	0.0276
Overall, VE by influenza type (A and B)	0.0021	0.0338
VE by age group – influenza A	0.0014	0.0340
VE by site – influenza A	0.0020	0.0482
VE by season – influenza A	0.0021	0.0495
VE by age group – influenza B	0.0021	0.0209
VE by site – influenza B	0.0036	0.0355
VE by season – influenza B	0.0039	0.0378
Overall, VE by influenza A subtype (H1N1pdm09 and H3N2)	0.0002	0.0050
VE by age group – influenza A (H1N1)pdm09	0.0069	0.0439
VE by site – influenza A (H1N1)pdm09	0.0108	0.0648
VE by season – influenza A (H1N1)pdm09	0.0088	0.0556
VE by age group – influenza A (H3N2)	NA*	0.0208
VE by site – influenza A (H3N2)	NA*	0.0405
VE by season – influenza A (H3N2)	NA*	0.0396

*b is not estimable and was fixed at 10^{-12}

© 2024 Grijalva CG et al. *JAMA Network Open*.

eTable 2. Vaccine Effectiveness Against Influenza A Infection Among Household Contacts, FluTES, Middle Tennessee and Central Wisconsin (2017-2020)

	Influenza A(H1N1)pdm09			Influenza A(H3N2)		
	Proportion of vaccinated contacts infected, n/N (%)	Proportion of unvaccinated contacts infected, n/N (%)	Adjusted Vaccine Effectiveness (95% CI)	Proportion of vaccinated contacts infected, n/N (%)	Proportion of unvaccinated contacts infected, n/N (%)	Adjusted Vaccine effectiveness (95% CI)
Overall	70/245 (28.6%)	90/261 (34.5%)	21.4 (-9.2, 43.5)	64/233 (27.5%)	42/190 (22.1%)	-26.9 (-91, 15.6)
Age group, n(%)						
<5 years	10/24 (41.7%)	15/25 (60.0%)	41.2 (-23.2, 72)	10/18 (55.6%)	3/7 (42.9%)	23.7 (-134.6, 75.2)
5-17 years	21/79 (26.6%)	37/100 (37.0%)	25.2 (-35.2, 58.6)	18/67 (26.9%)	22/76 (28.9%)	21.5 (-54.4, 60.1)
18-49 years	28/108 (25.9%)	31/115 (27.0%)	9.5 (-54.4, 47)	22/107 (20.6%)	15/89 (16.9%)	-58.9 (-216, 20.1)
>=50 years	11/34 (32.4%)	7/21 (33.3%)	-16.9 (-212.7, 56.3)	14/41 (34.1%)	2/18 (11.1%)	-176.7 (-1168.3, 39.6)
Site						
Central Wisconsin	48/153 (31.4%)	62/187 (33.2%)	1.4 (-47.1, 34)	25/114 (21.9%)	22/116 (19.0%)	-16.2 (-111.8, 36.2)
Middle Tennessee	22/92 (23.9%)	28/74 (37.8%)	46.4 (4.5, 69.9)	39/119 (32.8%)	20/74 (27.0%)	-16.5 (-107.6, 34.6)
Season						
2017-2018	1/7 (14.3%)	6/14 (42.9%)	82.6 (-51.7, 98)	15/82 (18.3%)	10/58 (17.2%)	4.2 (-126, 59.4)
2018-2019	22/86 (25.6%)	25/95 (26.3%)	9.6 (-63.4, 50)	49/151 (32.5%)	32/132 (24.2%)	-23.0 (-97.8, 23.5)
2019-2020	47/152 (30.9%)	59/152 (38.8%)	17.3 (-24, 44.9)	NA	NA	NA

Footnote: Overall Influenza type/subtype-specific vaccine effectiveness estimates were estimated using longitudinal chain binomial models, accounting for age group, site, household size, and season. Other vaccine effectiveness estimates accounted for the other variables included in the table, as appropriate. *Dash marker indicates estimates cannot be obtained due to zero events.

© 2024 Grijalva CG et al. *JAMA Network Open*.

eTable 3. Vaccine Effectiveness Against Influenza B Infection Among Household Contacts, FluTES, Middle Tennessee and Central Wisconsin (2017-2020)

	Proportion of vaccinated contacts infected, n/N (%)	Proportion of unvaccinated contacts infected, n/N (%)	Adjusted Vaccine Effectiveness (95% CI)
Influenza B, Victoria	21/141 (14.9%)	56/198 (28.3%)	49.5% (14.1%, 70.3%)
Influenza B, Yamagata	3/40 (7.5%)	8/55 (14.5%)	25.5% (-162.1%, 78.8%)

Footnote: Overall Influenza type/subtype-specific vaccine effectiveness estimates were estimated using longitudinal chain binomial models, accounting for age group, site, household size, and season.

© 2024 Grijalva CG et al. *JAMA Network Open*.

© 2024 Grijalva CG et al. *JAMA Network Open*.
