# Supplementary material for: Estimated Effectiveness of Influenza Vaccines in Preventing Secondary Infections in Households
Source: JAMA Netw Open. 2024 Nov 21;7(11):e2446814. doi: 10.1001/jamanetworkopen.2024.46814 (PMC11582933; doi:10.1001/jamanetworkopen.2024.46814)
Supplement: Supplement 2. — Data Sharing Statement [file jamanetwopen-e2446814-s002.pdf]

Data Sharing Statement

Grijalva. Estimated Effectiveness of Influenza Vaccines in Preventing Secondary Infections in Households. *JAMA Netw Open*. Published November 21, 2024.
doi:10.1001/jamanetworkopen.2024.46814

Data
Data available: No

Additional Information
Explanation for why data not available: The participants of this study did not give written consent for their data to be shared publicly, so study data are not available.
